# Nutrient Digestibility and Fecal Quality in Beagle Dogs Fed Meat and Bone Meal Added to Dry Food

**DOI:** 10.3390/vetsci9040164

**Published:** 2022-03-28

**Authors:** Amr Abd El-Wahab, Bussarakam Chuppava, Anna Lisa Zeiger, Christian Visscher, Josef Kamphues

**Affiliations:** 1 Institute for Animal Nutrition, University of Veterinary Medicine Hannover, Foundation, Bischofsholer Damm 15, D-30173 Hannover, Germany; amrwahab5@mans.edu.eg (A.A.E.-W.); sekretariat-tierernaehrung@tiho-hannover.de (A.L.Z.); christian.visscher@tiho-hannover.de (C.V.); josef.kamphues@tiho-hannover.de (J.K.); 2 Department of Nutrition and Nutritional Deficiency Diseases, Faculty of Veterinary Medicine, Mansoura University, Mansoura 35516, Egypt

**Keywords:** dog, meat bone meal, particle size, nutrient utilization, fecal score, fatty acids

## Abstract

Meat and bone meal (MBM) is one animal by-product used in pets. This study purposed to provide information on MBM including either coarsely (MBMc) or finely (MBMf) ground with regard to diet digestibility and fecal characteristics in dogs. Three different levels of MBM (6%, 12% and 24%) of each grinding form (MBM6, MBM12 and MBM24) were added to a basic diet. Six Beagle dogs (body weight 16.7 kg ± 0.42) participated in a Latin Square experiment. Each trial began with the animals adapting to the food for a five-day period, followed by five days of fecal collection. The feed particle size had no effect on the apparent digestibility of organic matter, crude protein and crude fat. The fecal score was significantly affected by the particle size × level interaction among treatments (*p* < 0.0001). It was noted that the different particle sizes or levels of MBM as main effects had no significant effect on the fecal fatty acid concentrations. These findings suggest that using coarse or fine grinding even including MBM up to 24% in dog diets does not affect the apparent digestibility of protein nor fecal quality negatively in our experimental study.

## 1. Introduction

Dogs require a high level of protein in their diet, which varies depending on many factors [1]. Adult and medium-sized dogs should have at least 18% crude protein, whereas puppies, pregnant and lactating dogs should have at least 25% [2]. Animal by-products means whole bodies or parts of animals, products of animal origin or other products obtained from animals, which are not intended for human consumption, including oocytes, embryos and semen according to European Union (EU) regulations [3]. The use of animal by-products in animal feed, including pet foods, is extensively regulated by the EU legislation, including Regulation 1069/2009 [4] and Regulation 142/2011 (animal by-products regulations) [3], Regulation 999/2001 [5] and Regulation 183/2005 on feed hygiene [6]. Meanwhile, in the EU, by-products account as much as 32, 38 and 46% of the body weight (BW) of chickens, pigs and cattle, respectively, and should be properly processed [7]. Currently, a very limited number of animal residues and derived products can be legally recycled to animal feed in the EU, i.e., only low risk category 3 animal by-products material, including expired food products, so-called ‘former foods’ not containing meat and fish [8]. Animal by-products before subsequent use in pet foods in the EU require a rendering process, as described for example by the European Pet Food Industry Federation (FEDIAF), with the exception of eligible former foods [9].

In accordance with the European Fat Processors and Renderers Association (EFPRA), which is one of Europe’s leading authorities on the safe disposal of animal fats and meat industry by-products, about 18 million metric tons are produced annually in the EU from slaughterhouses, food-producing plants for human consumption and dairies [8]. These residue materials are subsequently processed animal proteins account for about 2.5 million metric tons, and processed into about 4 million metric tons of animal fats and proteins [8]. For both the United States and Canada, over 200 rendering facilities have been documented [10], including both standalone rendering facilities and those incorporated into slaughterhouses that process up to 25 million metric tons per year [11]. Due to the fact that they offer the majority of the protein, fat and minerals in the diets, animal by-product meals have been (and continue to be) the key ingredient responsible for the worldwide pet food industry’s growth and expansion [12]. As a result, the processing steps required to obtain feed flour are gaining great attention [13,14].

Protein digestibility is of great concern in animal by-products. For instance, nutritionally, MBM remains a good source of animal-based protein [15], however, it can be reduced by excessive heating during MBM processing [16,17,18,19]. Nonetheless, heating is necessary because the safety control of MBM should be given special attention. The Association of American Feed Control Officers (AAFCO), for example, establishes and controls the implementation of these regulations in the United States [16]. In reality, within the animal feed sector, safety criteria for sources, processing and the use of rendered products have already been discussed [20]. The settings used to render animal by-products (133 °C at atmospheric pressure for 20 min) [21,22] are usually sufficient to eradicate pathogenic microorganisms present in the raw material.

On the other hand, it is generally recognized that grinding is a major concern during grain processing [23]. Raw material particle size influences nutrient digestibility in poultry and pigs [24,25]. However, little is known about the impact of raw material particle size on extruded dog diets. Indeed, in published studies, raw material particle size is either not specified or not fully determined [26,27,28,29].

MBM nutritional values reported in the literature vary greatly, most likely due to varying processing processes and raw material sources [30,31,32]. However, there is a lack of information on MBM digestibility and fecal characteristics in dogs (due to varied particle sizes and levels). Furthermore, to the best of our knowledge, investigations on the influence of particle size (coarse or fine) of MBM in dog diets on digestibility and fecal characteristics are extremely rare. Therefore, the purpose of our study was to determine the effects of increased proportions and varied particle sizes of MBM added to dry dog foods on nutrient digestibility as well as fecal quality.

## 2. Materials and Methods

Prior to conducting this investigation, the Animal Welfare Officer of the University of Veterinary Medicine Hannover, Foundation, Hannover, Germany evaluated and approved the study protocol in line with German regulation §7 of the Animal Protection Law (approval number TVO-2014-V-2).

### 2.1. Experimental Design

**Figure 1:**
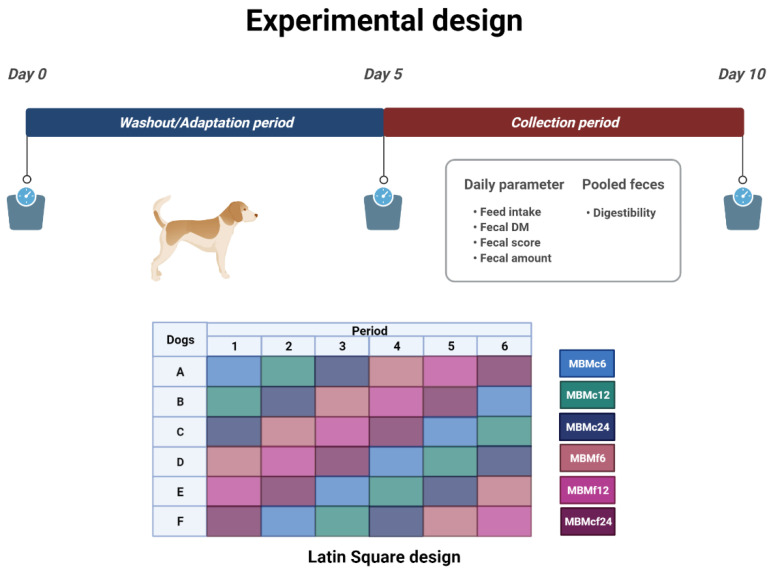
Concept of the Latin Square experimental design and parameters recorded during the collecting period (Figure was created with BioRender.com (accessed on 24 February 2022)).

The digestibility study conducted at the Institute for Animal Nutrition, University of Veterinary Medicine Hannover, Foundation included six healthy intact female Beagle dogs (*n* = 6). The University of Veterinary Medicine Hannover, Foundation provided all of the dogs used in this investigation. The dogs had a mean body weight (BW) of 16.7 kg ± 0.42 at the start of the trial, with a median age range of 6–10 years. According to Laflamme [33], the median body condition score during the entire experimental trial was 4.98 ± 0.312 out of 9. The trial was set up as a Latin Square design. Each trial began with the animals adapting to the food for a five-day period, followed by five days of fecal collection to determine individual apparent nutritional digestibility and fecal scores (Figure 1). Five days were used as wash-out before each experimental period (adaptation and collection) for each trial.

### 2.2. Diet Production

As a base meal, an extruded commercial dry diet (Fa. Fit + Fun Croc, MultiFit Tiernahrungs GmbH, Krefeld, Germany) containing cereals, meat and animal by-products, vegetable by-products, fats, oils and minerals (0.5%) was used as a basic diet (Table S1). The six experimental diets were created by adding coarse MBM or fine MBM to around 6%, 12% and 24% of the basic diet, respectively (MBMc6, MBMc12 and MBMc24 or MBMf6, MBMf12 and MBMf24, respectively). Briefly, for the groups MBM6 (6% replacement of basic diet by coarse MBM or fine MBM), the dogs were fed about 329 g fresh/d + 21 g of MBM/d. In the groups MBM12 and MBM24 (12% and 24% replacement of basic diets by coarse MBM or fine MBM), the dogs were fed about 308 g fresh/d + 42 g of MBM/d and 266 g fresh/d + 84 g of MBM/d, respectively.

### 2.3. Wet Sieve Analysis

The particle size distribution was determined for coarse and fine MBM (as an ingredient), a wet sieve analysis was conducted in accordance with Wolf et al. [34]. The sieves (sieve tower including with eight screen layers with the following mesh sizes in mm: 3.15; 2.0; 1.4; 1.0; 0.8; 0.56; 0.4; 0.2; Retsch GmbH, Haan, Germany) were firstly placed in hot air at 103 °C, and after cooling in a desiccator, were weighed separately. To ensure adequate hydration, a sample of approximately 30–50 g was soaked for one h in one L of water. Thereafter, the suspension was rinsed at a standardized pressure through the sieve tower with water (10 L). The wet sieves were dried at 103 °C overnight and weighed after cooling in a desiccator. The weight of particles retained in each sieve was then indicated as a percentage of the total dry matter (DM). The sample is washed out through the smallest screen layer (finest and dissolved components such as sugars and electrolytes) were calculated by subtraction.

### 2.4. Particle Size

The percentage of particle size distribution of MBMc and MBMf as ingredients varied markedly. About 37.4% of particles were >1.00 mm in MBMc vs. 7.50% in MBMf. Moreover, the particle size distribution <2.00 mm was about 19.1% in MBMc vs. 27.8% in MBMf.

### 2.5. Chemical Analysis

To determine the nutrients in the diets and fecal samples, the Association of German Agricultural Analytic and Research Institutes e.V. (VDLUFA) [35] procedures were used. Weighing the samples (about 50 g) before and after they were dried at 103 °C for 12 h yielded the dry matter (DM) content. Weighing the dried and ground samples (about 3 g) before and after combustion at 600 °C for 6 h was used to assess the crude ash content in the muffle furnace.

The total nitrogen content was also determined using the Dumas incineration method by placing about 0.3 g of the sample in a crucible at 1000 °C in an Elementar analyzer (Vario Max CNS, Elementar Analysensysteme GmbH, Langenfeld, Germany). The crude protein could then be determined by multiplying the nitrogen content by the factor 6.25.

The crude fat content was determined by acid digestion using the Soxhlet extraction method. Briefly, about 3 g of ground dry sample was mixed with 60 mL of hydrochloric acid and 140 mL distilled water for 30 min. Thereafter, the tube was filled up to 300 mL with distilled water and the contents filtered via a filter (595 ½ D 185 mm, Fa. Schleicher & Schuell Micro Science GmbH, Dassel, Germany). The filter was dried in a beaker overnight at 80 °C in a hot air oven. Subsequently, the fat was extracted with petroleum ether for 6 h in a standing flask, which was dried to constant weight in a Soxhlet extractor before this was distilled off again using a rotary evaporator (Rotavapor R114, Fa. Büschi, Switzerland). Finally, the flask was dried at 80 °C overnight and reweighed after cooling in a desiccator.

The crude fiber content was determined using diluted acidic and alkalic solutions and subsequent drying at 103 °C (Fibertec 2010 Hot Extraktor, Foss, Sweden). Briefly, 1 g of the ground material was weighed in a crucible and mixed with about 150 mL of 1.25% sulfuric acid for 30 min in the crude fiber determinator (Fibertec 2010 Hot Extraktor). After extracting the acid, this procedure was repeated with 1.25% sodium hydroxide solution. The crucible was then rinsed with hot distilled water, dried overnight at 103 °C and weighed after cooling in the desiccator. This was followed by drying the crucible in a muffle furnace at 500 °C for 2 h (ashing process), after which it was again weighed after cooling in the desiccator. The crude fiber content was calculated by subtracting the crude fiber content from the sample weight before ashing.

An atomic absorption spectrometer (Solaar M-Serie Atomic Absorption Spectometer, Thermo Elemental Ltd., Cambridge, England) was used to determine the calcium concentration in accordance with the Association of Official Analytical Chemists (AOAC) [36]. The phosphorus concentration was photometrically characterized (UV-Visible Recording Spectrophotometer UV 162, Schimadzu, Kyoto, Japan; Wavelength 356 nm) using the vanadate molybdate method, as described by Gericke and Kurmies [37]. Briefly, when adding orthophosphates to a reaction mixture consisting of ammonium molybdate, ammonium vanadate and nitric acid, a yellow compound is formed, which can be detected colorimetrically. About 10 mL of each of these reaction mixtures was transferred into a 50 mL flask. The negative sample was performed by ultrapure water, while the respective standards were filled up to the calibration mark with a defined amount of a phosphate stock solution with a phosphorus content of 1 mg/mL. Thereafter, the sample solution (max. 5000 µL) was added to the reaction mixture until the yellow coloration had reached the range between the standards. After all flasks had been filled, shaken and incubated for 30 min, the phosphorus content could finally be determined colorimetrically in the spectrophotometer.

Finally, ion-exchange chromatography (AA analyzer LC 3000, Biotronik Wissenschaftliche Geräte GmbH, Maintal, Germany) was used to analyze amino acid contents. The nitrogen-free extract content was calculated as follows: dry matter—(crude ash + crude protein + crude fat + crude fiber). Metabolizable energy (ME) content of the diets was calculated based on their chemical composition, as recommended by the National Research Council NRC, Washington, DC, USA [38].

### 2.6. Scores for Food Intake and Apparent Digestibility

The animals were fed once a day and given an unlimited supply of water. To calculate food intake, the amount of food offered and denied after each mealtime was recorded. Based on the energy need prediction equation for adult dogs following the metabolic weight [38], the amount of food supplied was estimated using a calculation based on their daily energy requirements (0.5 MJ ME BW^0.75^/d). The spontaneous acceptance “food intake assessment” (palatability and speed of food intake) was classified into three groups according to Zahn [39]. Briefly, score 1 = lowest acceptance (low palatable, refused intake at first offer, low amount of consumed food); score 2 = moderate acceptance (moderate palatable, hesitated intake at first offer, none to low amount of consumed food); and score 3 = maximum acceptance (high palatable, spontaneous intake at first offer, high amount of consumed food).

The apparent nutritional digestibility was determined using the whole fecal collection method [40], which included a five-day diet adaptation period followed by five-day fecal collection. Fresh feces were collected every day from the concrete floor during the collecting period. The DM content of a subsample of 10% of fresh feces per animal/d was evaluated after they had been weighed. The remaining fecal samples were then stored at −20 °C. The 5 d fecal samples from each dog were thawed, combined and homogenized at the end of the study. The apparent digestibility (percent) was computed by multiplying ((food − feces)/food) by 100 [41].

### 2.7. Fecal Quality

Every day, the amount of defecation was recorded. The feces were collected completely and individually every 30 min on five consecutive days (last five days of the ten-day period). Fecal consistency scores were noted on a five-point scale (1 = very hard, 2 = solid, well-formed “optimum”, 3 = soft, still formed, 4 = pasty, slushy and 5 = watery diarrhea) in accordance with Moxham [42]. A previous study [43] showed a photo of fecal consistency score.

### 2.8. pH Level

To determine the pH of fresh pooled feces on a daily basis, the samples were mixed 1:5 with distilled water, shaken, left at room temperature for 1 min and then measured with a pH meter (InLab^®^ Expert Pro, Mettler-Toledo International Inc., Columbus, OH, USA).

### 2.9. Volatile Fatty Acids

Fresh feces were obtained from each animal on the last day of the collection phase to determine fatty acids in accordance with Bunte et al. [44]. In summary, 1 g of feces was mixed with distilled water in a 1:5 ratio, rapidly agitated, then centrifuged at 4000 rpm for 15 min (Megafuge 1.0, Heraeus Deutschland GmbH & Co. KG, Hanau, Germany). After mixing the sample according to an internal standard (10 mL formic acid 89 percent and 0.1 mL 4-methylvaleric acid), the mixture was centrifuged before being exposed to gas chromatography (610 Series, Unicam Chromatography GmbH & Co. KG, Kassel, Germany) at 155 °C (injector: 175 °C, detector: 180 °C). The carrier gas was nitrogen, the flow rate was about 0.97 mL/min and the detector type was flame ionization in the gas chromatography.

### 2.10. Statistical Analysis

The statistical analysis was determined by using SAS^®^, version 9.3 of the Statistical Analysis System for Windows (SAS Institute, Inc., Cary, NC, USA). For all parameters, mean values as well as the standard deviation (SD) of the mean were computed. Each of the measured or recorded parameters was examined separately and served as the basis for the calculation. Data normality was verified using Univariate procedure. Normally distributed data, differences among treatments within each type of diet (coarse or fine) were determined using a Ryan–Einot–Gabriel–Welsch multi-range test (REGWQ test). For non-normally distributed data or values in the form of a score, the Kruskal–Wallis test was applied. In addition, data were analyzed as repeated measures data using the MIXED procedures of SAS. The model included feeding particle size, level of MBM and their interaction. *p* < 0.05 was used as the significant level. It has to be mentioned that the main effect, either particle size or level, was neglected if there was a particle size × level significant interaction effect.

## 3. Results

The general condition of dogs was healthy throughout the experimental period. The BW of the dogs was similar among the diet groups at the beginning of the study (*p* > 0.05) and did not change throughout the study (*p* > 0.05). Food intake scoring achieved a high level of acceptance scoring and no refusals were observed. All dogs consumed the total amount of the daily food offered to all groups (350 g as fed/dog).

### 3.1. Chemical Composition of Experimental Diets

The chemical analysis of MBMc ingredient revealed about 323, 551, 104, 0.00, 117 and 59.7 g/kg DM for crude ash, crude protein, crude fat, crude fiber, calcium and phosphorus levels, respectively. This contrasted with the chemical analysis of MBMf ingredients which revealed about 315, 571, 104, 0.00, 108 and 55.0 g/kg DM for crude ash, crude protein, crude fat, crude fiber, calcium and phosphorus levels, respectively.

The chemical composition of the experimental diets (Table 1) in this study varied considerably due to different levels and particle size of the MBM ingredient profiles. The amino acids profile of the MBM concerning ingredients is presented in Table S2. The DM content among the experimental canine foods was virtually similar (range: 918–924 g/kg). The crude ash, crude protein and crude fat contents increased linearly with an increasing inclusion level of MBMc (97.9; 112, and 141 g/kg DM crude ash; 228; 248 and 290 g/kg DM crude protein; 79.4; 80.9 and 84.1 g/kg DM crude fat for MBMc6, MBMc12 and MBMc24, respectively), while the level of crude fiber decreased with an increasing inclusion level of MBMc (27.2, 25.4 and 22.0 g/kg DM for MBMc6, MBMc12 and MBMc24, respectively). The same trend was observed with an inclusion of MBMf up to 24%. For example, the crude ash, crude protein and crude fat contents increased linearly with an increasing inclusion level of MBMf (97.4; 111 and 139 g/kg DM crude ash; 229; 251 and 294 g/kg DM crude protein, respectively). The levels of crude fat and crude fiber for MBMf6, MBMf12 and MBMf24 were identical to those for MBMc6, MBMc12 and MBMc24, respectively.

### 3.2. Apparent Nutrient Digestibility

The results of the apparent nutrient digestibility are presented in Table 2. Dogs fed coarsely or finely ground diets showed no significant differences in apparent nutrient digestibility. There were no effects of particle size on the digestibility of organic matter (range: 74.6–79.1% for MBMc; 72.9–78.2% for MBMf), crude protein (range: 82.3–83.1% for MBMc; 81.3–82.1% for MBMf) and crude fat (range: 87.2–88.0% for MBMc; 86.9–88.3% for MBMf). No significant effects of MBM levels were observed in the digestibility of organic matter (range: 82.5–84.4%), crude protein, (range: 81.3–83.1%) and crude fat (range: 86.9–88.3%) among the treatments. Additionally, digestibility of organic matter, crude protein and crude fat was not significantly affected by feed particle sizes on different levels of MBM (*p* = 0.6861, *p* = 0.8178 and *p* = 0.7945, respectively).

### 3.3. Fecal Quality

The data on fecal characteristics are presented in Table 3. The daily defecation frequency showed no significant differences among different particle sizes of MBM as a main effect (range: 2.60–3.20 for MBMc, and 2.53–3.37 for MBMf) and levels of MBM as a main effect (range: 2.53–3.37). No significant differences were noted in the defecation frequency by the particle size × level interaction effect among treatments (*p* = 0.1458). The amount of feces was significantly affected by the effect of particle size × level interaction among the treatments (*p* = 0.0192). The fecal score was significantly affected by the particle size × level interaction among treatments (*p* < 0.0001). The DM of fecal contents of feces was significantly affected among the treatments by the particle size × level interaction (*p* = 0.0011). Additionally, the pH values of feces were significantly affected among the treatments by the particle size × level interaction (*p* = 0.0003).

### 3.4. Volatile Fatty Acids

Table 4 shows the data from the fatty acid pattern in the feces of dogs fed different experimental diets. It was noted that the different particle size or levels of MBM as main effects had no significant effect on the fatty acid profiles. However, the content of iso-butyric acid in feces was significantly affected among treatments by the effect of particle size × level interaction (*p* = 0.0259). Additionally, only the contents of iso-valeric acid in feces were significantly affected among treatments by the effect of particle size × level interaction (*p* = 0.0396).

## 4. Discussion

A canine diet is composed of many factors that may affect nutrient digestibility, such as ingredient selection and nutrient composition [45,46]. The nutritional quality of meat and bone meal as potential sustainable sources of protein for pet food was evaluated in this study. A crucial point in the production of animal feeds is the particle size which determining both the nutritional quality of the diet and the processing efficiency [24]. Nevertheless, these aspects are little studied for dogs [47], and the data on the optimum particle size to extrude cereal-based dog foods are lacking. It was observed that including either coarsely or finely meat and bone meal in the dogs’ diets was well accepted and dogs consumed the total amount of the food offered. During the experimental study, the effects of protein sources in dog diets were investigated on apparent digestibility of nutrients as well as fecal characteristics.

### 4.1. The Digestibility of Nutrients

Generally, it is recognized that grinding is a major concern during grain processing [23]. The potential impact of rendering on nutrient digestibility was observed in poultry and pigs [24,25]. While in our study, the apparent digestibility of organic matter was not affected by particle size or the levels of MBM included in the diet. Moreover, the findings for dogs fed a coarsely or finely ground diet did not significantly differ between the levels of MBM in the diet. This is not in accordance with Zentek [48] and Bosch et al. [49] who stated that the apparent digestibility of organic matter was influenced by the amount of dietary protein. MBM is a heterogeneous mixture of particles; those derived from bone are high in ash, while those from soft tissues are high in protein. The primary protein in high-ash MBM is collagen; this protein is deficient in sulfur amino acids (cystine and methionine), tryptophan and isoleucine [32] and is poorly digested [50], which could affect the organic matter digestibility. However, in our study, the content of collagen was not determined and thus we cannot prove that it may play a role or not. Furthermore, no difference in organic matter digestibility was observed in the current study. Despite the crude protein content being higher in the diet, including a high level of MBM (range: 228–290 and 229–294 g/kg DM for MBMc and MBMf diets, respectively), we did not find any differences in organic matter digestibility between treatments.

Excessive temperatures during the processing systems have also been shown to negatively affect the protein digestibility of meat and bone meal [51]. An observation from the data in our study in dogs fed either coarse or fine MBM diets (range: 81.3–83.1%) showed a protein digestibility within the normal digestibility range (80%) described by the European Pet Food Industry Federation (FEDIAF) [9]. Interestingly, the structure seemed to have no influence on the protein digestibility. This is in agreement with an earlier study [52] that reported no effect on the degree of grinding in terms of protein digestibility. In the present study, the crude protein and crude fat contents were higher when increasing the MBM up to 24% compared to 6% of MBM (range: 290–294 and 84.1 g/kg as fed vs. 228–229 and 79.4 g/kg as fed, respectively). However, we found no difference regarding the apparent digestibility of crude protein across treatments. This is not in agreement with a previous study [53] whose authors concluded that high-protein diets may affect the digestibility due to undigested protein reaching the colon with the greater amounts. However, our results in the present study showed that no negative effect of MBM inclusion in dog food on protein digestibility was observed when increasing the ash content. In addition, dietary crude ash content is one factor that could affect protein digestibility. Despite the content of dietary crude ash being increased when raising the inclusion of MBM from 6% to 24% (97.9 vs. 141 and 97.4 vs. 139 g/kg DM for MBMc and MBMf, respectively), the apparent digestibility of crude protein was unaffected (range: 81.3–83.1%). This is in agreement with Johnson et al. [54] and Shirley and Parsons [55] described that increasing levels of ash in meat and bone meal have not been shown to lower protein digestibility. Contrary to our findings, Meyer and Mundt [52] stated that a higher crude ash content (up to 300 g/kg DM) in the food may leads to insufficient acidification of the chyme, which possibly result in lower protein digestibility.

According to NRC [38], fat is a key ingredient of MBM and therefore features as an energy source in pet diets. In the present study, when considering particle sizes or inclusion levels of the MBM diet offered to dogs, neither affected the apparent fat digestibility. The apparent fat digestibility was about 86.9–88.3% for MBM (coarse and fine). The fat content in our study increased when raising the inclusion of MBM in the diet (79.4 g/kg DM for 6% of MBM and 84.1 g/kg DM for 24% of MBM diets). The results of this study are in agreement with the findings of Hill et al. [56] who reported that the fat digestibility reached approximately 99% when dogs are fed diets containing a high amount of fat (about 320 g/kg DM). Additionally, Zuo et al. [57] observed that fat digestibility rose to about 97% when the amount of dietary fat rose. Overall, and based on the data in our study, the crude ash content in dog food does not contribute to fat digestibility.

### 4.2. Fecal Characteristics

Fecal quality is an important index used in the evaluation of dog foods. In the present study, fecal scores were maintained at acceptable levels, with a range of 1.82–2.61 for the MBMc diet and a range of 1.90–2.56 for MBMf. Increasing concentrations of MBM in the diet (regardless of particle size) resulted in a score of fecal consistency closer to the optimal value (score 2). Considering the interaction of the diet particle size and the inclusion level of MBM, dogs fed with high concentrations of MBM in the diet whether in coarsely or finely ground diets, the fecal consistency score was lower with a greater fecal DM as well as a higher pH value of feces was observed. Thus, the level of MBM in the diet influenced the fecal characteristic parameters, while the effect of particle size of the MBMs was not obvious. Therefore, a clearly positive influence of very high MBM inclusion (up to 24%) on fecal quality could be indicated under these experimental conditions. As reported in a previous study by Zentek et al. [58] which published an influence of the amount and type of protein source on the fecal quality, the fecal consistency with a good score was particularly related to a lower collagen content in the protein fraction of the food. The digestion and absorption of protein are considered to be one of the dietary factors affecting fecal DM content [59]. When protein is present but not absorbed, the dietary amino acids contained in that protein are unavailable for the dogs, and provide nitrogen substrate for proteolytic bacteria, which may result in reduced fecal quality [60]. Nery et al. [53] noted a softer fecal consistency at higher protein levels in dog food and stated that this could be explained by increased degradation of colonic fermentation processes. However, in the present study, the group fed MBM diets had a higher crude protein content in the MBM diets (+83–87 g/kg) but the fecal score was still good.

In the present study, feeding a diet with a high level of MBM with increasing concentrations of calcium, resulted numerically in reduced fecal scores, increased amounts of feces as well as an elevated fecal pH. Nevertheless, animal by-product meal with high-ash content, increased the content of calcium, phosphorus and magnesium, and reduced the digestibility of dry matter, which can lead to the formation of dry stools [61]. In contrast, Zanu et al. [62] stated that the fermentation of non-digested protein produces nitrogen compounds such as branched chain fatty acids (iso-butyrate and iso-valerate) [63] led to increased intestinal pH.

### 4.3. Fecal Volatile Fatty Acids

Volatile fatty acids (VFA) contribute to overall good health; individual VFA exert specific health benefits. For many years, the use of fecal short-chain fatty acids (SCFA) concentrations to estimate the fermentation activity of the microbiota of the large intestine of dogs has been practiced [64], and the microbiotas of dogs are active and have fermentation profiles similar in the rectum and the transversal colon [65]. However, McNeil et al. [66] determined that the SCFA produced are rapidly absorbed by the colon up to 95%, and that fecal analysis may not be the best response criterion reflecting the host animal’s SCFA status. In the present study, the fecal concentration of butyrate between different groups did not differ as there was a particle size × level significant interaction. Van der Steen et al. [67] observed that a high protein content in the diet led to an increased formation of iso-butyric acid and valeric acid. During the protein breakdown, valeric acid is formed at a low concentration and plays a role in the fermentation process of structural carbohydrates [67]. However, including MBM in the diet had no negative effects for the dogs due to the production of SCFA.

## 5. Conclusions

The results in the current study should be considered with caution due to the small number of animals. However, it is generally known that digestibility studies can be considered realistic even with a small number of dogs. The technology used in processing as well as the composition (ratio of, e.g., bone to meat in the MBM) can vary greatly between different companies and can affect the comparability of studies that have used similar products. Additionally, between batches of a company, products may vary in composition.

The sustainability of food animal production is greatly improved by recycling animal by-products such as meat and bone meal during the rendering process and using those by-products as feed ingredients for companion animals. It was observed in the current study that including either coarsely or finely ground MBM in the dog diets was well accepted and dogs consumed the total amount of the food offered. Apparent digestibility of protein was not affected when diets containing up to 24% coarsely or finely ground MBM were offered. Notably, it was possible to include MBM with up to 24% of either coarse or fine particle sizes without there being any negative effects on the fecal scores. Further studies are still required to investigate the effect of full replacement of the dietary protein sources by MBM on the nutrient digestibility and fecal quality of dogs, focusing also on the calcium to phosphorus ratio in the diet. Moreover, further research is still needed to determine the effect of particle size of different animal by-products on digestibility. Finally, it would be interesting to test other breeds in the future, for example German Shepherds that are known to have digestibility problems related to the characteristics of food.

## Figures and Tables

**Table 1:** Chemical composition of the basic diets supplemented with different levels and particle sizes of MBM.

Parameter	Unit	Basic	Coarse			Fine		
			MBMc6	MBMc12	MBMc24	MBMf6	MBMf12	MBMf24
Basic diet	%	100	94	88	76	94	88	76
MBMc		0	6	12	24	0	0	0
MBMf		0	0	0	0	6	12	24
DM	g/kg fed	924	923	921	918	924	923	922
Crude ash	g/kg DM	83.5	97.9	112	141	97.4	111	139
Crude protein		207	228	248	290	229	251	294
Crude fat		77.8	79.4	80.9	84.1	79.4	80.9	84.1
Crude fiber		28.9	27.2	25.4	22.0	27.2	25.4	22.0
Nitrogen-free extract		603	568	533	464	567	532	461
Calcium		21.6	27.3	33.1	44.5	26.8	32.0	42.3
Phosphorus		12.8	15.6	18.4	24.0	15.3	17.9	22.9
**Particle size**	%							
>1.00 mm		36.8	37.4			7.50		
0.20–1.00 mm		30.3	43.5			64.7		
<0.20 mm		32.9	32.9			27.8		
ME ^1^	MJ/100 g DM	15.6	15.6	15.6	15.7	15.6	15.7	15.7

^1^ Metabolizable energy (ME) content of the diets was estimated in accordance with NRC [38]. Sums of crude ash, crude fat, crude protein, crude fiber and N-free extracts may not total 1000 g due to rounding up.

**Table 2:** Apparent nutrient digestibility (%) of dogs fed basic diets supplemented with different levels and particle sizes of MBM (mean ± SD).

Parameters	Particle Size						*p*-Value		
	Coarse			Fine					
	Level						Particle Size	Level	Particle Size × Level
	MBMc6	MBMc12	MBMc24	MBMf6	MBMf12	MBMf24			
Organic matter	84.4 ± 2.03	83.8 ± 0.91	83.8 ± 0.81	83.7 ± 1.41	83.8 ± 3.08	82.5 ± 0.86	0.3059	0.7223	0.6861
Crude protein	82.3 ± 2.16	82.5 ± 1.19	83.1 ± 0.75	81.3 ± 2.28	81.5 ± 3.99	82.1 ± 1.47	0.2073	0.9493	0.8178
Crude fat	87.2 ± 2.10	87.2 ± 1.17	88.0 ± 1.23	87.4 ± 1.36	88.3 ± 2.50	86.9 ± 1.56	0.9714	0.6732	0.7945

MBM = Meat and bone meal, c = coarse, f = fine. No significant differences were noted among treatments, so that no superscripts were added.

**Table 3:** Fecal characteristics of dogs fed the basic diets supplemented with different levels and particle sizes of MBM (mean ± SD).

Parameters	Particle Size						*p*-Value		
	Coarse			Fine					
	Level						Particle Size	Level	Particle Size × Level
	MBMc6	MBMc12	MBMc24	MBMf6	MBMf12	MBMf24			
Defecation frequency (*n*/d)	2.60 ± 0.34	2.97 ± 0.57	3.20 ± 0.34	2.73 ± 0.70	2.53 ± 0.58	3.37 ± 0.95	0.8407	0.0904	0.1458
Amount of feces (g DM/d)	67.4 ^b^ ± 8.06	73.7 ^b^ ± 4.40	81.5 ^a^ ± 5.78	70.4 ^b^ ± 5.28	73.5 ^b^ ± 13.2	87.4 ^a^ ± 4.05	0.0800	0.0333	0.0192
Fecal score (1–5)	2.61 ^a^ ± 0.19	2.21 ^b^ ± 0.12	1.82 ^c^ ± 0.23	2.56 ^a^ ± 0.22	2.37 ^b^ ± 0.17	1.90 ^c^ ± 0.22	0.5985	<0.0001	<0.0001
DM content (%)	31.8 ^b^ ± 2.04	34.4 ^a^ ± 1.28	35.4 ^a^ ± 1.98	32.0 ^b^ ± 1.60	33.8 ^ab^ ± 1.76	36.2 ^a^ ± 2.10	0.8778	0.0005	0.0011
pH value	7.21 ^b^ ± 0.62	7.17 ^b^ ± 0.22	7.30 ^a^ ± 0.14	7.03 ^b^ ± 0.10	7.18 ^b^ ± 0.18	7.47 ^a^ ± 0.12	0.9863	0.0001	0.0003

MBM = Meat and bone meal, c = coarse, f = fine. ^a,b,c^ Means in a row with different superscripts differ significantly (*p* < 0.05). Fecal scores were recorded using a five-point scale (1 = very hard to 5 = watery diarrhea).

**Table 4:** Fatty acid profile in the feces (mmol/kg fresh feces) of dogs fed the basic diets supplemented with different levels and particle sizes of MBM (mean ± SD).

Parameters	Particle Size						*p*-Value		
	Coarse			Fine					
	Level						Particle Size	Level	Particle Size × Level
	MBMc6	MBMc12	MBMc24	MBMf6	MBMf12	MBMf24			
Acetic acid	23.4 ± 6.75	47.9 ± 35.0	32.0 ± 16.8	21.7 ± 14.2	27.1 ± 7.35	41.4 ± 23.8	0.5346	0.2561	0.1751
Propionic acid	10.2 ± 2.78	23.6 ± 16.5	13.5 ± 4.82	12.5 ± 10.3	12.3 ± 3.56	19.5 ± 10.7	0.7743	0.4182	0.1458
iso-Butyric acid	0.68 ^c^ ± 0.10	1.21 ^a^ ± 0.50	1.07 ^b^ ± 0.51	0.49 ^c^ ± 0.25	0.67 ^b^ ± 0.31	1.08 ^a^ ± 0.58	0.1269	0.0342	0.0259
n-Butyric acid	6.54 ± 5.53	17.4 ± 17.9	7.73 ± 6.84	6.19 ± 7.75	7.27 ± 5.32	5.99 ± 4.01	0.1990	0.3011	0.2613
iso-Valeric acid	0.99 ^c^ ± 0.13	1.69 ^a^ ± 0.68	1.54 ^ab^ ± 0.68	0.74 ^c^ ± 0.40	0.97 ^b^ ± 0.49	1.54 ^a^ ± 0.84	0.1352	0.0411	0.0396
n-Valeric acid	0.08 ± 0.10	0.20 ± 0.15	0.08 ± 0.05	0.11 ± 0.15	0.10 ± 0.09	0.05 ± 0.05	0.3438	0.4962	0.2769

MBM = Meat and bone meal, c = coarse, f = fine. ^a,b,c^ Means in a row with different superscripts differ significantly within each grinding (*p* < 0.05).

## Data Availability

The data presented in this study are available in this manuscript and the Supplementary Materials.

## Supplementary Materials

The following are available online at https://www.mdpi.com/article/10.3390/vetsci9040164/s1, Table S1: Ingredient composition and additives (per kg) of the basic diet. Table S2: Levels of amino acids in the experimental raw ingredients (g/kg DM).

## References

[1] Vanelli K., de Oliveira A.C.F., Sotomaior C.S., Weber S.H., Costa L.B. (2021). Soybean meal and poultry offal meal effects on digestibility of adult dogs diets: Systematic review. PLoS ONE.

[2] Federação Europeia das Indústrias de Food (FEDIAF) (2017). Nutritional Guidelines.

[3] European Commission (EC) (2011). Commission Regulation (EU) No 142/2011 of 25 February 2011 implementing Regulation (EC) No 1069/2009 of the European Parliament and of the Council laying down health rules as regards animal by-products and derived products not intended for human consumption and implementing Council Directive 97/78/EC as regards certain samples and items exempt from veterinary checks at the border under that Directive Text with EEA relevance. Off. J. Eur. Union.

[4] European Commission (EC) Regulation (EC) No 1069/2009 of the European Parliament and of the Council of 21 October 2009 Laying Down Health Rules as Regards Animal By-Products and De-Rived Products not Intended for Human Consumption and Repealing Regulation (EC) No. 1774/2002. http://eur-lex.europa.eu/legal-content/EN/ALL/?uri=CELEX%3A32009R1069.

[5] European Commission (EC) (2001). Regulation (EC) No 999/2001 of the European Parliament and of the Council of 22 May 2001 laying down rules for the prevention, control and eradication of certain transmissible spongiform encephalopathies. Off. J. Eur. Union.

[6] European Commission (EC) (2005). Regulation (EC) No 183/2005 of the European Parliament and of the Council of 12 January 2005 laying down requirements for feed hygiene. Off. J. Eur. Union.

[7] Alm M. Review of the EU feed ban on non-ruminant Processed Animal Proteins. Proceedings of the European Fat Processors and Renderers Association (EFPRA).

[8] Jedrejek D., Lević J., Wallace J., Oleszek W. (2016). Animal by-products for feed: Characteristics, European regulatory framework, and potential impacts on human and animal health and the environment. J. Anim. Feed Sci..

[9] The European Pet Food Industry Federation (FEDIAF) (2018). Nutritional Guidelines for Complete and Complementary Pet Food for Cats and Dogs.

[10] Jekanowski M. (2011). Survey says: A snapshot of rendering. Render.

[11] Meeker D.L., Meisinger J.L. (2015). Companion Animals Symposium: Rendered ingredients significantly influence sustainability, quality, and safety of pet food. J. Anim. Sci..

[12] Corbin J.E., Pearson A.M., Dutson T.R. (1992). Inedible Meat, Poultry and Fish By-Products in Pet Foods. Inedible Meat by-Products.

[13] Batalov A., Luneva R., Gorelik O. Methods of Production of Meat-and-Bone Meal According to New Technologies. http://min.usaca.ru/uploads/article/attachment/1744/42_%D0%91%D0%B0%D1%82%D0%B0%D0%BB%D0%BE%D0%B2_%D0%90.%D0%A1.pdf.

[14] Mushtruk M.M. Analytical Review of Technologies and Equipment for Production of Feed Flour and Its Mixtures. https://www.researchgate.net/profile/Mikhail-Mushtruk-2/publication/328756194-ANALYTICAL_REVIEW_OF_TECHNOLOGIES_AND_EQUIPMENT_FOR_PRODUCTION_OF_FEED_FLOUR_AND_ITS_MIXTURES/links/60be28c6458515218f9edacf/ANALYTICAL-REVIEW-OF-TECHNOLOGIES-AND-EQUIPMENT-FOR-PRODUCTION-OF-FEED-FLOUR-AND-ITS-MIXTURES.pdf.

[15] Parsons C., Castanon F., Han Y. (1997). Protein and amino acid quality of meat and bone meal. Poult. Sci..

[16] Simonova I., Grabovskyi S., Drachuk U., Halukh B., Basarab I. (2020). Amino acid composition of meat and bone meal from various manufacturers of pet food and animal feed. Ukr. J. Ecol..

[17] Hao H., Cheng G., Iqbal Z., Ai X., Hussain H.I., Huang L., Dai M., Wang Y., Liu Z., Yuan Z. (2014). Benefits and risks of antimicrobial use in food-producing animals. Front. Microbiol..

[18] Rey I.U., Shakulikova G.T., Kozhakhmetova G.A., Lashkareva O.V., Bondarenko E.G., Bermukhambetova B.B., Baimagambetova Z.A., Zhetessova M.T., Beketova K.N., Anafiyaeva Z. (2016). Labor Factor Efficiency in the Agricultural Industry. Int. J. Environ. Sci. Educ..

[19] Mizanbekova S., Umbetaliev A., Aitzhanova A., Akylbaev R. (2017). Priorities of Mixed Fodder Production Development in Emerging Countries: The Case of Kazakhstan. Espacios.

[20] Woodgate S., Van Der Veen J. (2004). The role of fat processing and rendering in the European Union animal production industry. Biotechnol. Agron. Soc. Environ..

[21] European Commission (EC) Regulation (EC) No 1774/2002 of the European Parliament and of the Council of 3 October 2002 Laying Down Health Rules Concerning Animal By-Products not Intended for Human Consumption. http://eur-lex.europa.eu/legal-content/EN/TXT/PDF/?uri=CELEX:32002R1774&from=ES.

[22] OIE Bovine Spongiform Encephalopathy, Volume II. Recommendations Applicable to OIE Listed Diseases and Other Diseases of Importance to International Trade. http://www.oie.int/fileadmin/Home/eng/Health_standards/tahc/current/chapitre_bse.pdf.

[23] Bazolli R.S., Vasconcellos R.S., de-Oliveira L.D., Sá F.C., Pereira G.T., Carciofi A.C. (2015). Effect of the particle size of maize, rice, and sorghum in extruded diets for dogs on starch gelatinization, digestibility, and the fecal concentration of fermentation products1. J. Anim. Sci..

[24] Wondra K., Hancock J., Behnke K., Hines R., Stark C. (1995). Effects of particle size and pelleting on growth performance, nutrient digestibility, and stomach morphology in finishing pigs. J. Anim. Sci..

[25] Amerah A.M., Ravindran V., Lentle R.G., Thomas D.G. (2007). Feed particle size: Implications on the digestion and performance of poultry. Worlds Poult. Sci. J..

[26] Carciofi A.C., Takakura F.S., De-Oliveira L.D., Teshima E., Jeremias J.T., Brunetto M.A., Prada F. (2008). Effects of six carbohydrate sources on dog diet digestibility and post-prandial glucose and insulin response. J. Anim. Physiol. Anim. Nutr..

[27] Twomey L.N., Pluske J.R., Rowe J.B., Choct M., Brown W., Pethick D.W. (2003). The replacement value of sorghum and maize with or without supplemental enzymes for rice in extruded dog foods. Anim. Feed Sci. Technol..

[28] Twomey L.N., Pethick D.W., Rowe J.B., Choct M., Pluske J.R., Brown W., Laviste M.C. (2002). The use of sorghum and corn as alternatives to rice in dog foods. J. Nutr..

[29] Fortes C.M.L.S., Carciofi A.C., Sakomura N.K., Kawauchi I.M., Vasconcellos R.S. (2010). Digestibility and metabolizable energy of some carbohydrate sources for dogs. Anim. Feed Sci. Technol..

[30] Adedokun S.A., Adeola O. (2005). Metabolizable energy value of meat and bone meal for pigs. J. Anim. Sci..

[31] Olukosi O., Adeola O. (2009). Estimation of the metabolizable energy content of meat and bone meal for swine. J. Anim. Sci..

[32] Wang X., Parsons C.M. (1998). Effect of raw material source, processing systems, and processing temperatures on amino acid digestibility of meat and bone meals. Poult. Sci..

[33] Laflamme D.P. (1997). Development and validation of a body condition score system for dogs. Canine Pract..

[34] Wolf P., Rust P., Kamphues J. (2010). How to assess particle size distribution in diets for pigs?. Livest. Sci..

[35] Naumann C., Bassler R. (2012). Methoden der Landwirtschaftlichen Forschungs-und Untersuchungsanstalt, Biochemische Untersuchung von Futtermitteln.

[36] Association of Official Analytical Chemists (AOAC) (2000). Official Methods of Analysis.

[37] Gerickend S., Kurmies B. (1952). Die kolorimetrische Phosphorsäuerebestimmung mit Ammonium-Vanadat-Molybdat und ihre Nawendung in der Pflanzenanalyse. Pflanzenernähr. Dünger Bodenk..

[38] National Research Council (NRC) (2006). Nutrient Requirements of Dogs and Cats.

[39] Zahn S. (2010). Untersuchungen zum Futterwert (Zusammensetzung, Akzeptanz, Verdaulichkeit) und zur Verträglichkeit (Kotbeschaffenheit) von Nebenprodukten der Putenschlachtung bei Hunden. Doctoral Thesis.

[40] Association of American Feed Control Officials (AAFCO) (2014). Model Regulations for Pet Food and Specialty Pet Food under the Model Bill.

[41] Kamphues J., Wolf P., Coenen M., Eder K., Iben C., Kienzle E., Liesegang A., Männer K., Zebeli Q., Zentek J. (2014). Supplement zur Tierernährung für Studium und Praxis.

[42] Moxham G. (2001). Waltham feces scoring system—A tool for veterinarians and pet owners: How does your pet rate. Waltham Focus.

[43] Abd El-Wahab A., Wilke V., Grone R., Visscher C. (2021). Nutrient Digestibility of a Vegetarian Diet with or without the Supplementation of Feather Meal and Either Corn Meal, Fermented Rye or Rye and Its Effect on Fecal Quality in Dogs. Animals.

[44] Bunte S., Keller B., Chuppava B., Kamphues J., Visscher C., El-Wahab A.A. (2020). Influence of Fermented Diets on In Vitro Survival Rate of Some Artificially Inoculated Pathogens—A Preliminary Study. Processes.

[45] Earle K.E., Kienzle E., Opitz B., Smith P.M., Maskell I.E. (1998). Fiber affects digestibility of organic matter and energy in pet foods. J. Nutr..

[46] Gilani G.S., Xiao C.W., Cockell K.A. (2012). Impact of antinutritional factors in food proteins on the digestibility of protein and the bioavailability of amino acids and on protein quality. Br. J. Nut..

[47] Hilcko K.P., Félix A.P., de Oliveira S.G., Bortolo M., Maiorka A., de Brito C.B.M., Alves P.F. (2009). Diferentes graus de moagem em dietas para cães. Ciência Rural.

[48] Zentek J. (1996). Cellulose, pectins and guar gum as fibre sources in canine diets. J. Anim. Physiol. Anim. Nutr..

[49] Bosch G., Zhang S., Oonincx D.G., Hendriks W.H. (2014). Protein quality of insects as potential ingredients for dog and cat foods. J. Nutr. Sci..

[50] Ravindran V., Hendriks W.H., Camden B.J., Thomas D.V., Morel P.C.H., Butts C.A. (2002). Amino acid digestibility of meat and bone meals for broiler chickens. Aust. J. Agric. Res..

[51] Batterham E., Lowe R., Darnell R., Major E. (1986). Availability of lysine in meat meal, meat and bone meal and blood meal as determined by the slope-ratio assay with growing pigs, rats and chicks and by chemical techniques. Br. J. Nutr..

[52] Meyer H., Mundt H. (1983). Untersuchungen zum Einsatz von Knochenschrot in Futterationen fur Hunde. Dtsch. Tierarztl. Wochenschr..

[53] Nery J., Goudez R., Biourge V., Tournier C., Leray V., Martin L., Thorin C., Nguyen P., Dumon H. (2012). Influence of dietary protein content and source on colonic fermentative activity in dogs differing in body size and digestive tolerance. J. Anim. Sci..

[54] Johnson M.L., Parsons C.M., Fahey G.C., Merchen N.R., Aldrich C.G. (1998). Effects of species raw material source, ash content, and processing temperature on amino acid digestibility of animal by-product meals by cecectomized roosters and ileally cannulated dogs. J. Anim. Sci..

[55] Shirley R., Parsons C. (2001). Effect of ash content on protein quality of meat and bone meal. Poultr. Sci..

[56] Hill R., Burrows C., Ellison G., Bauer J. (2001). The effect of texturized vegetable protein from soy on nutrient digestibility compared to beef in cannulated dogs. J. Anim. Sci..

[57] Zuo Y., Fahey G.C., Merchen N., Bajjalieh N. (1996). Digestion responses to low oligosaccharide soybean meal by ileally-cannulated dogs. J. Anim. Sci..

[58] Zentek J., Kaufmann D., Pietrzak T. (2002). Digestibility and effects on fecal quality of mixed diets with various hydrocolloid and water contents in three breeds of dogs. J. Nutr..

[59] Weber L.W., Boll M., Stampfl A. (2004). Maintaining cholesterol homeostasis: Sterol regulatory element-binding proteins. World J. Gastroenterol..

[60] Hall J.A., Melendez L.D., Jewell D.E. (2013). Using gross energy improves metabolizable energy predictive equations for pet foods whereas undigested protein and fiber content predict stool quality. PLoS ONE.

[61] Murakami F.Y., de Lima D.C., Menezes Souza C.M., Kaele G.B., de Oliveira S.G., Félix A.P. (2018). Digestibility and palatability of isolated porcine protein in dogs. Ital. J. Anim. Sci..

[62] Zanu H.K., Kheravii S.K., Bedford M.R., Swick R.A. (2020). Dietary calcium and meat and bone meal as potential precursors for the onset of necrotic enteritis. Worlds Poult. Sci. J..

[63] Musco N., Lombardi P., Calabrò S., Mastellone V., Tudisco R., Grossi M., Addi L., Grazioli R., Cutrignelli M.I. (2016). Aloe arborescens supplementation in cat diet: Evaluation of effects by in vitro gas production technique. Ital. J. Anim. Sci..

[64] Swanson K.S., Grieshop C.M., Flickinger E.A., Bauer L.L., Chow J., Wolf B.W., Garleb K.A., Fahey G.C. (2002). Fructooligosaccharides and Lactobacillus acidophilus modify gut microbial populations, total tract nutrient digestibilities and fecal protein catabolite concentrations in healthy adult dogs. J. Nutr..

[65] Bosch G., Pellikaan W.F., Rutten P.G.P., van der Poel A.F.B., Verstegen M.W.A., Hendriks W.H. (2008). Comparative in vitro fermentation activity in the canine distal gastrointestinal tract and fermentation kinetics of fiber sources. J. Anim. Sci..

[66] McNeil N.I., Cummings J., James W. (1978). Short chain fatty acid absorption by the human large intestine. Gut.

[67] Van der Steen I., Rohde J., Zentek J., Amtsberg G. (1997). Fütterungseinflüsse auf das Vorkommen und die Enterotoxinbildung von Clostridium perfringens im Darmkanal des Hundes. Kleintierprax.

