# Supplementary material for: Nutrient Digestibility and Fecal Quality in Beagle Dogs Fed Meat and Bone Meal Added to Dry Food
Source: Vet Sci. 2022 Mar 28;9(4):164. doi: 10.3390/vetsci9040164 (PMC9031195; doi:10.3390/vetsci9040164)
Supplement: Supplementary file 1 [file vetsci-09-00164-s001.zip › vetsci-1638086-supplementary.pdf]

veterinary sciences

MDPI

Supplementary Table S1. Ingredient composition and additives (per kg) of the basic diet.

Ingredient	Unit	
Grains		-†
Meat and animal by-products		-
Vegetable by-products		-
Oils and fats		-
Minerals		-
Vitamin A	IU	11500
Vitamin D3		1150
Zinc	mg	70
Copper (copper-(II)-sulphate, pentahydrate)		10
Iodine (calcium iodate, anhydrous)		2
Selenium (sodium selenite)		0.2

† An extruded commercial dry diet (Fa. Fit+Fun Croc, MultiFit Tiernahrungs GmbH, Krefeld, Germany), the values were not available.

Supplementary Table S2. Levels of amino acids in the experimental raw ingredients (g/kg DM).

Amino Acid	MBM	
	Coarse	Fine
Aspargine	45.0	45.9
Threonine	19.4	20.0
Serine	21.5	21.4
Glutamine	78.0	78.3
Glycine	71.1	71.3
Alanine	42.3	43.3
Valaline	22.7	20.9
Cysteine	5.10	4.60
Methionine	9.83	9.72
Ileucine	18.3	17.3
Leucine	34.0	33.9
Tyrosine	13.4	13.0
Phenylalanine	19.4	20.0
Histadine	11.1	11.0
Lysine	34.5	35.4
Arganine	42.9	45.8
Proline	46.1	48.3

MBM = Meat bone meal. Amino acid contents were calculated.
